# Systematic Evaluation of the Accelerate Pheno System for Susceptibility Testing of Gram-Negative Bacteria Isolated from Blood Cultures

**DOI:** 10.1128/Spectrum.01836-21

**Published:** 2021-12-22

**Authors:** Yera A. Patel, Thomas J. Kirn, Melvin P. Weinstein, Priyanka Uprety

**Affiliations:** a ProHealth Care, Division of Infectious Disease, Optum, Lake Success, New York, USA; b Department of Pathology and Laboratory Medicine, Department of Medicine, Rutgers Robert Wood Johnson Medical School, New Brunswick, New Jersey, USA; Weill Cornell Medicine

**Keywords:** rapid phenotypic antimicrobial susceptibility test, Accelerate Pheno, Gram-negative, blood cultures

## Abstract

Bacteremia is a major cause of morbidity and mortality. Rapid identification of pathogens for early targeted antimicrobial therapy is crucial for detecting emergence of antibiotic resistance and improving outcomes. However, there are limited data regarding the analytical performance of a rapid identification (ID) and antimicrobial susceptibility testing (AST) method like Accelerate Pheno blood culture detection system compared with the conventional methods routinely used in microbiology laboratories. We undertook a systematic quality improvement (QI) study to compare AST results obtained with Accelerate Pheno system rapid ID/AST system with a standard reference method in a university hospital microbiology laboratory. This was a single center, retrospective (5/10/19 to 8/1/19) and prospective (8/1/19 to 1/31/20) study that evaluated all blood cultures growing Gram-negative rods (GNR). We compared AST results obtained using the reference disk diffusion (DD) susceptibility method with those obtained by the Accelerate Pheno system. We calculated the error rates and categorical agreement between the Accelerate Pheno system and DD for each organism and specific drug tested. We evaluated 355 blood cultures growing GNR, of which 284 met the inclusion criteria. We grouped all Enterobacterales (*n*  =  263) for analysis (156 Escherichia coli, 60 Klebsiella spp., 20 Proteus mirabilis, 17 Enterobacter spp., and 10 Serratia marcescens*)*. Twenty-one Pseudomonas aeruginosa isolates were analyzed separately. For Enterobacterales, categorical agreement (CA) was ≥90% for amikacin (AMK), aztreonam (ATM), cefepime (FEP), ceftriaxone (CRO), ertapenem (ETP), gentamicin (GEN), meropenem (MEM), and tobramycin (TOB); and very major error (VME) was <5% for ampicillin/sulbactam (SAM), GEN, MEM, TOB, CRO, and ceftazidime (CAZ). For ciprofloxacin (CIP), CA was 87% and VME was 8%. For P. aeruginosa, CA was ≥90% for AMK and TOB, and VME was ≥5% for AMK, CAZ, GEN, MEM, piperacillin-tazobactam (TZP), and TOB. Accelerate Pheno rapid ID/AST system for GNR isolated from blood culture (BCs) was reliable for some but not all agents in the panel. Based on the findings from this study, our laboratory reports Accelerate Pheno system AST results only for Enterobacterales, and we limit our reports to CRO, CAZ, TZP, CIP, ATM, and GEN.

**IMPORTANCE** This was an 8-month retrospective and prospective study looking at the analytical performance of the Accelerate Pheno system on clinical isolates obtained from patients seen in our tertiary care hospital. Most of the published literature on the analytical performance of Accelerate Pheno System has been from clinical trials with limited data from clinical microbiology laboratories postimplementation of the system. Here we compare the AST results on 355 blood cultures growing Gram-negative bacteria in Accelerate Pheno system with the CLSI reference disk diffusion (DD) method. The findings from this study highlight the “real-world” performance of the Accelerate Pheno system for Gram-negative bacteria from blood cultures. We provide data to show the reliable susceptibility testing results of Enterobacterales for most of the commonly used antimicrobial agents and significant limitation for susceptibility testing results of Pseudomonas aeruginosa on the Accelerate Pheno system.

## INTRODUCTION

Bacteremia is a major cause of morbidity and mortality, and its increasing incidence is complicated by the challenge of antimicrobial resistance. Rapid identification (ID) of pathogens for early targeted antimicrobial therapy is crucial for reducing mortality, emergence of antibiotic resistance, and improving outcomes (1–3) . Empiric antimicrobials often are initiated while results from conventional methods for identification and antimicrobial susceptibility testing (AST) are pending, a process that may take up to 48 h.

The Accelerate Pheno system is a diagnostic instrument used to identify bloodstream pathogens from signal positive blood cultures in approximately 2 h and provide phenotypic susceptibility results in about 7 h. The Accelerate Pheno system is an automated system that follows a series of processes. It starts with gel electrofiltration which separates impurities such as salt, extracellular debris, and proteins from the bacterial cells; the bacterial cells are then immobilized by electrokinetic concentration. This then allows for identification of the organism using the fluorescence *in situ* hybridization (FISH) method by identifying specific nucleic acid sequences which are then compared with universal bacterial probe signals (https://acceleratediagnostics.com/products/accelerate-pheno-system/). The MIC susceptibility is done using a sample concentration and dynamic dilution method which automatically dilutes to a target range, avoiding use of manual McFarland preparation. Then, using morphokinetic cellular analysis, individual cells and colonies are identified and their division and growth patterns are analyzed and interpreted based on breakpoints for the respective organism and antimicrobial agent.

Studies have been done at multiple medical centers comparing the Accelerate Pheno system to the respective institutions standard of care (4, 5). One study done at two medical centers found that the Accelerate Pheno system was able to provide ID and AST of organisms faster than a standard of care method by more than a day. Overall, it was fairly accurate but unable to replace the current methods as there were a significant number of instances where Accelerate Pheno recommended performing alternative methods to confirm the instrument’s results (4). At our institution, we noted occasional discrepancies with both identification and susceptibility results between the laboratory’s existing methods and the Accelerate Pheno system. These have included lack of either an identification or a susceptibility result with the Accelerate Pheno system, incorrect/inconsistent identification, and nonreproducible antimicrobial susceptibility results. To address our concerns related to susceptibility results more systematically, we compared Accelerate Pheno susceptibilities with the CLSI reference disk diffusion (DD) method. Herein, we describe the analytical performance of Accelerate Pheno system compared with the DD susceptibility method.

## RESULTS

A total of 355 blood cultures growing Gram-negative bacteria were evaluated, of which 284 met the inclusion criteria and 71 were excluded (isolates not available [21], polymicrobial [7], no ID [26], no AST results from the Accelerate Pheno instrument [17]). Of the 284 Gram-negative susceptibility results analyzed, 92.6% (*n*  =  263) were Enterobacterales (Escherichia coli*, n*  =  156; Klebsiella spp., *n*  =  60; Proteus mirabilis, *n*  =  20; Enterobacter spp., *n*  =  17; and Serratia marcescens, *n*  =  10), and 7.4% were Pseudomonas aeruginosa (*n*  =  21).

For Enterobacterales (Table 1), categorical agreement (CA) was ≥ 90% for ceftriaxone, cefepime, ertapenem, meropenem, aztreonam, gentamicin, tobramycin, and amikacin. CA was 88% for ceftazidime and piperacillin-tazobactam, 87% for ciprofloxacin and 83% for ampicillin/sulbactam. The Accelerate Pheno system error rates for Enterobacterales are also shown in Table 1. Very major error (VME) rates were ≤3% for meropenem, gentamicin, and tobramycin; 4% for ceftazidime, 5% for ceftriaxone, 7% for aztreonam, 8% for ciprofloxacin, and >10% for piperacillin-tazobactam, cefepime, ertapenem, and amikacin. Major error rates were ≤3% for all drugs tested except ampicillin-sulbactam (14%).

**TABLE 1:** Diagnostic accuracy of Accelerate Pheno blood culture detection system for Enterobacterales (*n*  =  263)^*a*^

Antibiotics	Categorical agreement		VME		ME		mE	
	*N*	%	*N*	%	*N*	%	*N*	%
Amikacin	261	97	3	60	1	0.4	4	2
Ampicillin-sulbactam	235	83	2	3	2	2	37	16
Aztreonam	262	93	2	7	3	1	13	5
Cefepime	263	90	4	13	3	1	20	8
Ceftazidime	263	88	1	4	10	4	21	8
Ceftriaxone	263	94	2	5	3	1	11	4
Ciprofloxacin	262	87	7	8	0	0	26	10
Ertapenem	262	97	3	60	2	1	2	1
Gentamicin	263	99	0	0	1	0.4	2	1
Meropenem	240	98	0	0	1	0.4	4	2
Piperacillin-tazobactam	261	88	1	11	6	3	25	10
Tobramycin	263	93	1	3	2	1	16	6

a Enterobacterales (*n*  =  263), 156 E.coli, 60 Klebsiella spp., 20 Proteus mirabilis, 17 Enterobacter, and 10 S. marcescens. VME, very major errors; ME, major errors; mE, minor errors.

For P. aeruginosa (Table 2), CA was ≥90% for tobramycin and amikacin, 81% for gentamicin, 80% for meropenem, 79% for cefepime, 76% for ciprofloxacin, and <40% for piperacillin-tazobactam and ceftazidime. VME rates were ≤3% for piperacillin-tazobactam, ceftazidime, meropenem, and the aminoglycosides but were high for cefepime (50%) and ciprofloxacin (40%). Major errors (ME) were ≤3% for all of the drugs tested except cefepime (9%), piperacillin-tazobactam (19%), and ceftazidime (67%).

**TABLE 2:** Diagnostic accuracy of Accelerate Pheno blood culture detection system for Pseudomonas aeruginosa (*n*  =  21)^*a*^

Antibiotics	Categorical agreement		VME		ME		mE	
	*N*	%	*N*	%	*N*	%	*N*	%
Amikacin	21	100	0	0	0	0	0	0
Cefepime	14	79	1	50	1	9	1	7
Ceftazidime	12	25	0	0	6	67	3	25
Ciprofloxacin	21	76	2	40	0	0	3	14
Gentamicin	21	81	0	0	0	0	4	19
Meropenem	20	80	0	0	0	0	4	20
Piperacillin-tazobactam	21	38	0	0	3	19	10	48
Tobramycin	21	95	0	0	0	0	1	5

a VME, very major errors; ME, major errors; mE, minor errors.

## DISCUSSION

The results of this study show both the potential utility and current limitations of the Accelerate Pheno system to provide early phenotypic susceptibility results for patients with Gram-negative bacteremia. As used in our busy university hospital clinical microbiology laboratory, the Accelerate Pheno system provided excellent (>90%) CA for Enterobacterales versus amikacin, gentamicin, tobramycin, aztreonam, ceftriaxone, cefepime, ertapenem, and meropenem and CA of 87% to 88% for piperacillin-tazobactam, ceftazidime, and ciprofloxacin. CA was poor for ampicillin-sulbactam. However, because there were relatively few resistant isolates, the number of VME was unacceptably high for many of the drugs tested. VME was <3% only for meropenem and gentamicin. VME for ceftriaxone and ceftazidime were 5% and 4%, respectively, and VME for tobramycin was 3%. VME for ampicillin-sulbactam was 3%, for which overall CA was poor. In contrast, three of five Enterobacterales that were resistant to amikacin by reference DD testing were susceptible by Accelerate Pheno (VME = 60%). Major errors for Enterobacterales tested by Pheno were mostly <3%; antimicrobials with ME <3% included ceftriaxone, ertapenem, meropenem, aztreonam, amikacin, gentamicin, and tobramycin. The ME rates for piperacillin-tazobactam, ceftazidime, and ampicillin sulbactam were 3%, 4%, and 2%, respectively. When tested versus Enterobacterales, ampicillin-sulbactam, piperacillin-tazobactam, and ciprofloxacin and very high minor error rates (16%, 10% and 10%, respectively). Performance of the antipseudomonal β-Lactams for P. aeruginosa on the Accelerate Pheno system was suboptimal with high major error rates for cefepime (9%), piperacillin-tazobactam (19%), and ceftazidime (67%). Our results are similar to that from the multicenter study of the Accelerate Pheno system with a major error rates of cefepime (13%), piperacillin-tazobactam (2.9%), and ceftazidime (24%) (5). Our study showed a high (50%) VME for ceftazidime for P. aeruginosa. Result from the multicenter study of the Accelerate Pheno system showed no VME for 25 resistant P. aeruginosa (5). However, our results cannot be directly compared with the multicenter study as our study had few (*n*  =  2) resistant P. aeruginosa isolates to draw a meaningful conclusion.

Accurate and rapid phenotypic susceptibility results for Gram-negative bacteria other than Enterobacterales, primarily P. aeruginosa, is essential for appropriate clinical management of patients. Phenotypic AST is particularly challenging for P. aeruginosa due to its growth characteristic (6). Only limited classes of antimicrobial agents can be used to treat pseudomonal bacteremia due to its intrinsic resistance, and ongoing emergence of multi-drug-resistance organism in health care setting (7). Hence, it is imperative to obtain rapid and accurate phenotypic results for this group of organisms to guide appropriate antimicrobial agent for clinical management. Recent updates made to the Accelerate PhenoTest BC kit and software reportedly have improved the analytic performance of beta-lactams versus P. aeruginosa (8). A study conducted by Accelerate Diagnostics using 144 P. aeruginosa challenge isolates showed improved performance for cefepime (0% VME, 0% ME, 14.7% minor error (mE)), piperacillin-tazobactam (0% VME, 0% ME, 5.8% mE), and ceftazidime (3.2% VME, 1% ME, 5% mE). Testing in our study was done using the older blood culture kit (1.4.3.1) and previous version of the software (1.4.1.28). Our laboratory has not yet adopted the new Accelerate PhenoTest BC kit and the new version of the software (1.4.1.25) that showed improved performance for antipseudomonas drugs that were used in the study by Sikorski et al. (8).

One of the major limitations of this study is that it was a single center evaluation and there were small numbers of certain organisms, most notably P. aeruginosa, for which the available data were too limited to draw conclusions regarding routine implementation of the Accelerate Pheno system. We could also not perform rigorous studies for verification of VME, mainly for P. aeruginosa due to a very limited number of clinical isolates that were resistant. Nevertheless, the data presented here were from clinical isolates obtained from patients seen in our tertiary care hospital. Another significant limitation is that we were only able to use the DD reference method for comparison and not an MIC reference method, and we were only able to calculate categorical agreement between the methods. Lastly, we were also not able to determine the accuracy of less frequently encountered organisms such as Acinetobacter baumannii.

In conclusion, the Accelerate Pheno system may provide a potential step forward in rapid identification and phenotypic susceptibility testing from positive blood cultures. In our laboratory, the system identified most Gram-negative bloodstream isolates (data not shown) and was reliable for susceptibility testing of Enterobacterales for some but not all commonly used antimicrobial agents.

## MATERIALS AND METHODS

This was a single center, retrospective (5/10/19 to 8/1/19) and prospective (8/1/19 to 1/31/20) study that evaluated all blood cultures growing Gram-negative rods at Robert Wood Johnson University Hospital (RWJUH), a 610-bed tertiary care hospital in New Brunswick, New Jersey. Blood cultures were collected in BD Bactec Aerobic and Anaerobic media and incubated on Bactec FX (Becton, Dickinson and Co., Franklin Lakes, NJ, USA) instruments for up to 5 days. Blood cultures that grew only Gram-negative bacteria were set up on the Accelerate Pheno system (Accelerate Diagnostics, Tucson, AZ, USA) following manufacturer’s recommendations. Cultures that grew Gram-positive bacteria, mixed organisms, or yeasts were excluded per laboratory protocol. All positive blood cultures were plated to 5% sheep blood, chocolate, and MacConkey agars. Routine identification of the organism was done by matrix-assisted laser desorption/ionization (MALDI)-time of flight (TOF) mass spectrometry (MS) (Bruker Daltonics, Bremen, Germany) and antimicrobial susceptibility testing was done by the reference DD susceptibility method (9).

A total of 355 blood cultures growing Gram-negative bacteria were evaluated in the 8-month study period. Susceptibility results from the Accelerate Pheno system were compared with the CLSI reference DD susceptibility method. CLSI breakpoints from the 28th edition were applied (10) . For study purposes, the categorical AST result (susceptible [S], intermediate [I], resistant [R]) obtained using the reference DD susceptibility method was compared with the categorical result obtained by the Accelerate Pheno system. CA rates were calculated, and discrepancies were further evaluated by calculating error rates: VME, ME, and mE for each organism-antibiotic combination. VME, or falsely susceptible, were defined as susceptible by Accelerate Pheno and resistant by DD. ME, or falsely resistant, were defined as resistant by Accelerate Pheno and susceptible by DD. mE were defined an intermediate result by one method and susceptible or resistant result by the other method. Categorical agreement was defined as the percentage of isolates with the same categorical result (S, I, or R).
